# Effects of breeding center, age and parasite burden on fecal triiodothyronine levels in forest musk deer

**DOI:** 10.1371/journal.pone.0205080

**Published:** 2018-10-01

**Authors:** Xiaolong Hu, Yuting Wei, Songlin Huang, Gang Liu, Yihua Wang, Defu Hu, Shuqiang Liu

**Affiliations:** 1 Laboratory for Non-invasive Research Technology for Endangered Species, College of Nature Conservation, Beijing Forestry University, Beijing, China; 2 China Wildlife Mark Center, Chinese Academy of Forestry, Beijing, China; 3 Institute of Wetland Research, Chinese Academy of Forestry, Beijing, China; Institute of Animal Science, CZECH REPUBLIC

## Abstract

The objective of this study was to evaluate the effects of sex, breeding center and age on fecal triiodothyronine levels in captive forest musk deer *Moschus berezovskii*, and to explore the age-intensity model of gastrointestinal parasites. Furthermore, the association between fecal triiodothyronine levels and parasite egg shedding was also analyzed. We collected musk deer fecal samples from two breeding centers located in Shaanxi and Sichuan province, China. Enzyme-linked immunosorbent assays were utilized to estimate the fecal triiodothyronine concentrations and profiles, and fecal parasite eggs or oocysts were counted using the McMaster technique. Female deer from both breeding centers consistently showed higher triiodothyronine concentrations than those observed in males, which indicates that a distinct physiology pattern occurs by sex. The triiodothyronine concentration in Sichuan breeding center was significantly higher than that in Shaanxi center for both sexes, suggesting that differences in environment, diet and management practices are likely to affect the metabolism. In addition, a negative relationship between triiodothyronine concentrations and age was found (r = - 0.75, p < 0.001), and parasite egg shedding was also negatively associated with age (r = - 0.51, p < 0.001), by which we can infer that older animals evolves a more developed immune system. Finally, a positive association between parasite egg shedding and triiodothyronine levels was found, which could be explained by the additional energy metabolism resulting from parasitic infection. Results from this study might suggest metabolic and immunological adaptations in forest musk deer. These baseline data could be used to unveil metabolic status and establish parasite control strategies, which has great potential in captive population management as well as their general health evaluations.

## Introduction

Thyroid hormones are important regulators of metabolism, growth, development, reproduction, and homeostasis in mammals and birds [1, 2]. Thyroid hormones include triiodothyronine (T3), reverse triiodothyronine (rT3), thyroxine (T4), and reverse thyroxine (rT4), and of these the most potent component is T3 [3, 4]. Levels of thyroid hormones are affected by several factors, including nutrition [5, 6], temperature [7], age [6, 8], sex [9, 10], and reproduction [8, 11]. Parasite infection poses serious threats to animal health, and creates burdens on the host physiological metabolism [12]. The adaptive secretion of thyroid hormones is an important response mechanism to metabolic disorders and adverse environments. However, few studies have analyzed the associative pattern between gastrointestinal (GI) parasite infection and thyroid hormones.

Several studies have examined the relationship between haematozoon infection and thyroid hormones [13, 14], and found that these parasites cause a decrease in serum T3 levels. The life cycle and pathogenesis of the haematozoon are distinct from GI parasites. How GI parasites interact with T3 is still unclear. The infection of GI parasites leads to emaciation and dyspepsia, which is similar to the clinical symptoms of thyroid disorders [15]. The analysis of associative patterns between GI parasite infection and fecal T3 levels can facilitate the health management of captive endangered species, and help us to explore the adaptive strategies of hosts responding to GI parasite infections.

Forest musk deer (FMD, *Moschus berezovskii*) is a small ruminant that inhabits forests and mountains of East Asia, with China as the most common distribution area [16]. Overexploitation and habitat destruction have resulted in a sharp decline both in the population size and distribution of wild FMD, prompting the establishment of breeding centers within the original habitats [17]. Nevertheless, the population size of breeding FMD remains very small, partly due to the highly sensitive physiology inherent to this species and high rates of parasitic infection [18, 19]. Meanwhile, disease diagnosis and preventive treatment require knowledge of baseline levels of metabolic physiology of FMD, which has been lacking due to the conservation status of this species and limited sample availability. The baseline data of fecal T3, reflecting an individual’s metabolic characteristics, could be used to unveil its physiological status and identify individuals with abnormal physiological status. Usually, blood is considered as the best source for measuring circulating levels of thyroid hormones. However, it is difficult to obtain blood samples from endangered wildlife, and capturing FMD for blood collection may induce anxiety in these animals. Recent studies have attempted to measure thyroid hormone levels in feces [20], and T3 appears to be the more informative thyroid hormone in feces compared to T4 [21].

The aim of this present study was to investigate fecal T3 levels of captive FMD to determine basal concentrations and trends among various age classes. At the same time, a comparative analysis was performed to investigate the effects of breeding center, diet and management practice on T3 concentrations. Finally, age-related differences in parasite egg shedding were explored, and the associative pattern between parasite egg shedding and fecal T3 levels was investigated.

## Materials and methods

### Study areas and animals

All fecal samples were collected from the Shaanxi and Sichuan FMD breeding centers, China. The Shaanxi breeding center is located in Hanzhong (33°35′N, 106°49′E), Shaanxi Province, on the south slope of the Qinling Mountains, and situated at an altitude of 1,500 m. The Sichuan breeding center is located in Aba (34°11′N, 106°50′E), Sichuan Province, east of the Tibetan Plateau, at an altitude of 2,800 m. All animals were fed with leaves collected from their natural habitats twice daily at 7:00 h and 18:00 h. FMD at the Sichuan center were fed with *Usnea diffracta*, *Lactuca sativa*, *Brassica oleracea var*. *capitata*, whereas the feed of the Shaanxi center included *Anacardiaceae rhus*, *Morus alba*, *Simaroubaceae picrasma*, and *Ulmus pumila*. The supplementary feed of the two centers was the same, including a mixture of soybean flour, wheat bran, corn flour, and seasonal fruits. The FMD were kept together during the day but separated at night, so feces could be collected from each animal.

### Sample collection

A total of 101 adult individuals from the Shaanxi center and 120 adult individuals from the Sichuan center were selected for sampling from July 1 to August 10, 2014 (S1 Table). To control for the unstable physiology of captive FMD, we collected 10 samples from each animal for a more accurate determination of fecal T3. Samples from each animal were collected every 4 days, thereby generating a total of 2210 fecal samples for hormonal analysis. Subsequently, 204 fresh fecal specimens for parasitological analysis were collected from the 68 males in Sichuan center, and each animal was sampled once daily and continuously for 3 days. The feces from all individual stalls were cleaned every evening from 18:00 to 20:00 h, thereby allowing the collection of fresh feces from each musk deer the next day at 7:00 h. The birth year of all animals born at the center were recorded by breeders, and ear tags were used to distinguish musk deer, so we can know their age. The animals in age 3–4 were grouped into age group 3, and so on. The samples for hormonal analysis were frozen at– 20°C and transported to our laboratory in a mobile refrigerator. The samples for parasitological analysis were preserved in 10% formalin solution. All selected animals were presumed healthy and showed no abnormal conditions during the research period.

The authors assert that all procedures contributing to this work comply with the ethical standards of the relevant national and institutional guides on the care and use of laboratory animals. The fecal sampling was carried out under the authority of a scientific permit issued by the Shaanxi and Sichuan Forestry Bureau, China. The non-invasive sampling method was used to collect feces only.

### Extraction and measurement of triiodothyronine levels

Fecal T3 was extracted as previously described, with minor modifications [21]. Up to 2 g of feces was homogenized and freeze-dried prior to extraction to allow hormone concentrations to be expressed per gram of dry weight, while controlling for possible variations due to diet and variable moisture levels. Approximately 15 mL of 70% ethanol was added to 0.1 g of freeze-dried and thoroughly homogenized feces, vortexed for 10 min, incubated in a water bath at 60°C for 20 min, and then centrifuged for 20 min at 2,500 rpm. The supernatant was decanted into a tube, and the fecal pellet was re-extracted (10 mL, 70% ethanol) a second time. The supernatants were then pooled and dried in a water bath at 60°C, re-dissolved in 1 mL methanol, and stored at—20°C until analysis.

Enzyme-linked immunosorbent assays (ELISA) were used to quantify fecal T3 concentrations. The ELISA analyzer was a Spark 10M (TECAN, Switzerland), and the corresponding quantitative diagnostic kits (Bovine Triiodothyronine ELISA Kit, Cusabio, China) were used to determine T3 levels. We selected Bovid species-specific kit rather than the Cervidae species-specific kit, because *Moschus* species was phylogenetically related to bovid rather than to cervids [22]. The assays were performed according to the directions provided by the manufacturers. The detection range for T3 was 0.1–0.8 ng/mL, and the sensitivity of the kits was ≤ 0.2 ng/mL. The intra-assay coefficient of variation was < 10%, and inter-assay coefficient of variation was < 15%. The previous study has performed parallelism and accuracy studies to validate the T3 assay on Moose [21], indicating that fecal extracts were not interfering with the measurement precision. The parallelism test was used to validate the ELISA: the serial dilution ratios of 1:32, 1:16, 1:8, 1:4, 1:2, and 1:1 were performed, and standard curve were obtained to compare the slope with F-tests, and the non-significant differences indicated good assay parallelism.

### Parasitological analysis

The mean eggs per gram (EPG) or oocysts per gram (OPG) in fecal samples over three days were counted using the McMaster technique [23], and the parasite egg shedding was defined as the summation of EPG or OPG of all parasite species in each sample [24]. The microscopic analysis was performed within 2 weeks of sample collection in accordance with our previous study [19] with minor revision: 2g of feces were ground up thoroughly, mixed with 58ml of saturated sodium chloride and stirred continuously for 20 min until the feces was homogenized thoroughly. Each mixture was filtered into a new beaker through a standard sieve with 0.15 mm mesh, and the resulting filtrate was injected into two counting chambers of a McMaster Egg Slide Counting Chamber (Shanghai Veterinary Research Institute, Chinese Academy of Agricultural Sciences). Microscopy was performed after the eggs or oocysts were floating for 5 min. The EPG or OPG were calculated as: EPG or OPG = (n / 0.15) × V / m, where n is the mean number of eggs or oocysts in two counting chambers and 0.15 is the volume of each counting chamber, whereas V and m are the volume of the homogenized fecal sample and weight of feces, respectively; in this case V = 60 ml and m = 2 g.

### Data analysis

Hormonal data for each animal was obtained after removing outliers using an iteration process as follows: values above/below mean values ± 1.5 standard deviations were considered as outliers, and means were recalculated until all outliers were excluded (S1 Table). The normality of data was tested using the Kolmogorov–Smirnov test, finding that the T3 data was normal. The parasitic data was Log (x + 1) transformed to meet parametric assumptions (p > 0.05). Independent samples *t*-tests showed that T3 concentrations did not differ significantly between lactating and non-lactating females (S2 Table). Consequently, data of lactating and non-lactating females within each age group was pooled for further statistical analyses. We tested for effects of sex, age and breeding center on T3 concentrations and parasite egg shedding by constructing multivariate Generalized Linear Models (GLMs), with T3 and egg shedding as the dependent variables, and sex, age, breeding center and their interactions as the predictor variables. Pairwise comparison (independent-samples *t*-test for testing sex and breeding center, one-way ANOVA for testing age) was performed if statistically significant factors or interactions were detected. The correlations between T3 and age, parasite egg shedding and age, and parasite egg shedding and T3 were determined using Spearman’s correlation analyses. The significance threshold was 0.05 (α = 0.05), and the sequential Holm-Bonferroni correction was used to control Type I error. All statistical analyses were performed with SPSS version 20.0 (IBM Corporation, Armonk, NY, USA).

## Results

### Associations between T3 levels and sex, breeding center, and aging

The GLM revealed that sex (χ^2^ = 94.79, df = 1, p < 0.001), breeding center (χ^2^ = 76.26, df = 1, p < 0.001) and age (χ^2^ = 408.92, df = 3, p < 0.001) all showed significant effects on fecal T3 levels, whereas there was no significant interaction among these three factors (p > 0.05; Table 1). The females from both breeding centers showed significantly higher T3 levels than males (*t*-test, p < 0.05; Table 2). The FMD from Sichuan breeding center showed significantly higher T3 levels than those from Shaanxi center (*t*-test, p < 0.05; Table 2). The age-related differences in fecal T3 concentrations were significant at Shaanxi breeding center (female, F = 30.55, p < 0.001; males, F = 28.59, p < 0.001; Table 2), and also at Sichuan breeding center (female, F = 82.11, p < 0.001; males, F = 25.55, p < 0.001; Table 2). Meanwhile, the Spearman correlation analysis revealed significantly negative correlations between T3 concentration and age in FMD from both Shaanxi (female, r = - 0.86, p < 0.001; male, r = - 0.78, p < 0.001) and Sichuan breeding center (female, r = - 0.90, p < 0.001; male, r = - 0.72, p < 0.001). The highest T3 concentrations were found in deer with 3 years of age (134.00 ± 1.47 ng/g), whereas deer with 9 years of age showed the lowest concentration (101.53 ± 1.23 ng/g). The significances of differences in fecal T3 concentrations among age classes are presented in S3 Table.

**Table 1:** The effects of sex, age, breeding center and their interactions on the fecal triiodothyronine (T3) and parasite egg shedding of forest musk deer using multivariate Generalized Linear Models (GLMs).

Dependent variables	Sources	co-efficient value (χ^2^)	df	p value
T3	Breeding center	76.26	1	<0.001
	Sex	94.79	1	<0.001
	Age	408.92	3	<0.001
	Breeding center*Sex	0.05	1	0.832
	Breeding center*Age	0.69	3	0.877
	Sex*Age	1.92	3	0.590
	Breeding center*Sex*Age	0.06	3	0.997
Parasite egg shedding	Age	10.80	3	0.001

**Table 2:** Pairwise comparison (independent-samples *t*-test) of effects of sex, age and breeding center on fecal T3 levels (mean ± SE) of forest musk deer.

Years of age	Shaanxi center (ng/g)		Sichuan center (ng/g)	
	Male	Female	Male	Female
3	121.28 ± 2.24	134.52 ± 3.78	132.01 ± 2.11	144.89 ± 1.42
5	110.85 ± 2.24	122.83 ± 2.65	121.74 ± 2.51	134.22 ± 2.32
7	100.54 ± 1.67	111.83 ± 1.24	110.85 ± 3.66	122.83 ± 1.12
9	93.30 ± 2.64	100.80 ± 0.30	101.19 ± 1.18	110.33 ± 1.26

### Relationships between age and parasite egg shedding

**Fig 1:**
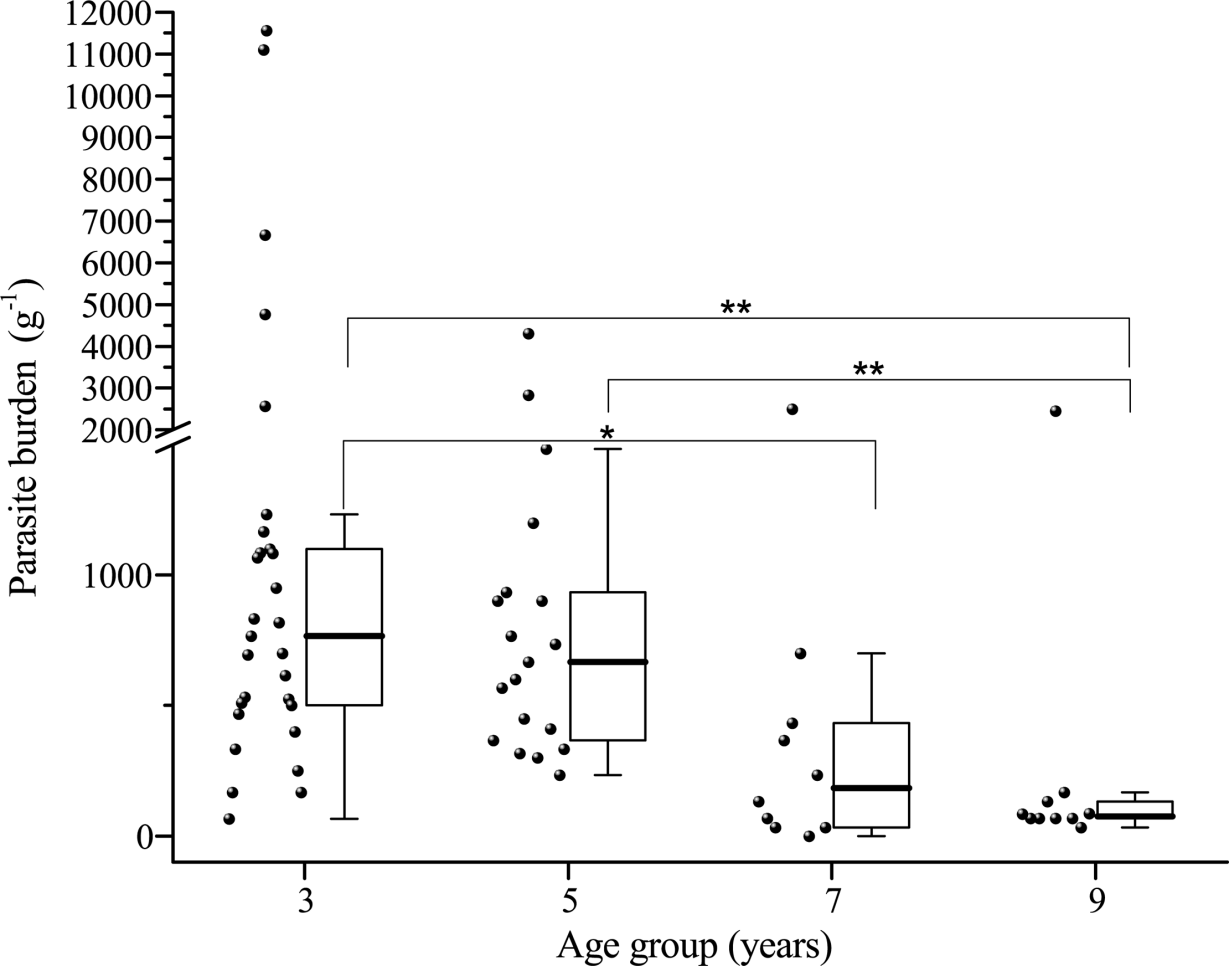
Age-related differences in parasite shedding of forest musk deer. * indicates that a significant difference (p < 0.05) was detected, and ** represents an extremely significance (p < 0.01). The significances were determined by General Linear Model.

The age-related differences in parasite egg shedding were significant (GLMs, χ^2^ = 10.80, df = 3, p = 0.001, Table 1), and Spearman correlation analysis indicated a significantly negative correlation between parasite shedding and age levels (r = - 0.51, p < 0.001). The parasite egg shedding in group of 3 years of age was significantly higher than those 7 years of age (F = 10.72, p < 0.001) and 9 years of age (F = 10.72, p < 0.001), and the group of 5 years of age also showed markedly higher parasite egg shedding than those 7 years of age (F = 10.72, p = 0.001) and 9 years of age (F = 10.72, p < 0.001; Fig 1). The highest egg shedding was found in deer with 3 years of age (1817.17 ± 553.15), whereas the group of 9 years of age group showed the lowest egg shedding (322.20 ± 236.73).

### Association between parasite egg shedding and fecal T3 levels

Fecal T3 levels were positively correlated with parasite egg shedding both in 3 years of age (r = 0.62, p < 0.001) and 5 years of age (r = 0.55, p = 0.015). However, no significant correlations were found in 7 years of age (r = 0.43, p = 0.221) and 9 years of age (r = - 0.39, p = 0.267).

## Discussion

Thyroid hormone levels tended to be sex biased, with higher T3 concentrations found in females than males, regardless of the breeding center and age. This trend is in agreement with those reported in dolphins *Tursiops truncatus* [25], Rhesus monkeys *Macaca mulatta tcheliensis* [26], pigs *Susscrofa domestica* [27], Moghani sheep *Ovis aries irania* [28], and white goat *Capra aegagrus hircus* [11]. The sex-biased thyroid hormone levels are partly due to the differences in the metabolic physiology and reproduction mechanism between the female and the male. In mammalian species, thyroid hormones are essential for the maintenance of female reproductive behaviors (e.g. sustain pregnancy and raise offspring) [29]. Our study found that lactating and non-lactating females showed similar T3 levels, whereas the baseline levels of T3 in females were higher than in males, which might indicate a higher energy consumption of female in breeding seasons than males. Furthermore, T3 levels may also be related to the activity of animals, and a previous study [21] reported that more rapid fecal excretion of T3 occurs in more active dogs. Female FMD are always allowed to wander within the enclosures every day, whereas males are only allowed outside occasionally.

The FMD from Sichuan breeding center showed significantly higher T3 levels than those from Shaanxi center. Temperature is one of the important regulatory environmental factors of thyroid function, i.e. the stimulation of lower temperatures causes an increase in T3 levels [11]. The Sichuan breeding center has an average temperature of 16.3°C in summer, compared with 26.1°C in the Shaanxi breeding center. In a low temperature environment, the homeotherm needs to generate more heat to keep their body warm, which consequently increases their energy metabolism [30, 31]. We hypothesize that this factor may explain some of the observed differences by breeding center. Furthermore, differences in diet and breeding management are likely contributed to variances observed between two breeding centers [18]. In the present study, T3 levels significantly declined as the age increased, which are consistent with other reports in rats *Rattus norvegicus* [32], humans *Homo sapiens* [33, 34], sea lions *Eumetopias jubatus* [6], sheep *Ovis aries* [8], and dolphins *Tursiops truncatus* [35]. Thyroid hormones play a key role in coordinating different factors involved in growth. In addition, they directly influence growth by altering biochemical reactions, cause positive nitrogen balance, and promote growth and development [36].

Parasite egg shedding was negatively correlated with age. The present results are consistent with several studies revealing significantly higher parasite egg shedding in feces of young animals compared to older animals or adults [37–40]. This may be related to the lower immunity in young animals [41]. This study found a positive relationship between parasite infection and fecal T3 levels in deer of 3 years of age and 5 years of age, rather than in 7 years of age and 9 years of age. One possible reason is that the immune system in older animals is more developed, which could allow it to respond to parasite infection before initiating the pathway of T3-based metabolism. Several studies have clearly reported adaptive immune responses to intestinal parasites in older animals [42, 43].

Studies on *Theileria annulata* [13] and *Babesia gibsoni* [14] reported that T3 concentrations are significantly lower in hosts infected with these parasites than in the healthy hosts. The tick-borne diseases such as babesiosis and theileriosis could result in systemic inflammatory response syndrome and consequently dysfunction in multiple organs [44]. In contrast, the GI parasites have a symbiotic relationship with the hosts, and the survival of parasites have to be dependent on the nutritional intake of the host. Thus, the hosts have to increase their food intake for complementing the extra energy requirement resulting from GI parasite infection, and then enhance their own energy metabolism to compete with parasites for nutrients.

## Conclusions

The present study indicates a noteworthy relationship between biotic (sex, age, parasite burden) and abiotic (breeding center) factors with T3 levels in FMD. Meanwhile, the sex-related differences in T3 levels reflect distinct metabolic physiology between female and male FMD. The negative relationship between parasite egg shedding and age suggests an immune adaption with the aging of hosts. The results have great potential in future management of FMD and relative ruminants at several aspects: 1) the baseline information on fecal thyroid hormones with bovine T3 antibody has been established, which can be used to unveil physiological status and metabolic characteristics of FMD; 2) when breeders feed FMD, they should take consideration of factors of sex, age and even breeding center to guide the diet allocation, for example, the younger FMD should be given the diet containing more concentrate food to meet the higher energy demand; 3) the results have revealed a relationship between thyroid hormones and GI parasite infection, which may be a general pattern in ruminants, in turn may potentially improve techniques in disease diagnosis.

## Supporting information

S1 Table Number of forest musk deer used for collecting fecal samples from different breeding centers and age groups. Numbers in the brackets represent the number of valid data after removing the outliers.
(DOCX)

S2 Table Comparison between lactating and non-lactating females for each age groups at Shaanxi and Shaanxi breeding center. The significances were determined using the independent-samples *t*-test.
(DOCX)

S3 Table The significances of age-related differences in fecal T3 levels of musk deer at Shaanxi and Sichuan breeding center. The significances were determined using the one-way ANOVA.
(DOCX)

## References

[1] Oppenheimer JH. Evolving concepts of thyroid hormone action. Biochimie. 1999; 81: 539–543. 10403187 10.1016/s0300-9084(99)80107-2

[2] Mullur R, Liu YY, Brent GA. Thyroid hormone regulation of metabolism. Physiol Rev. 2014; 94: 355–382. 10.1152/physrev.00030.2013 24692351 PMC4044302

[3] Tomasi TE. Utilization rates of thyroid hormones in mammals. Comp Biochem Phys A. 1991; 100: 503–516. 10.1016/0300-9629(91)90363-h 1685967

[4] Norman AW, Litwack G. Hormones 2nd ed California: Academic Press; 1997.

[5] Kohel KA, MacKenzie DS, Rostal DC, Grumbles JS, Lance VA. Seasonality in plasma thyroxine in the desert tortoise, *Gopherus agassizii*. Gen Comp Endocrinol. 2001; 121: 214–222. 10.1006/gcen.2000.7595 11178887

[6] Myers MJ, Rea LD, Atkinson S. The effects of age, season and geographic region on thyroid hormones in Steller sea lions (*Eumetopias jubatus*). Comp Biochem Physiol A. 2006; 145: 90–98. 10.1016/j.cbpa.2006.05.004 16815718

[7] Kahl S, Elsasser TH, Rhoads RP, Collier RJ, Baumgard LH. Environmental heat stress modulates thyroid status and its response to repeated endotoxin challenge in steers. Domest Anim Endocrinol. 2015; 52: 43–50. 10.1016/j.domaniend.2015.02.001 25804834

[8] Novoselec J, Antunović Z, Šperanda M, Steiner Z, Šperanda T. Changes of thyroid hormones concentration in blood of sheep depending on age and reproductive status. Ital J Anim Sci. 2010; 8: 208–210.

[9] Morganti S, Ceda GP, Saccani M, Milli B, Ugolotti D, Prampolini R, et al Thyroid disease in the elderly: sex-related differences in clinical expression. J Endocrinol Invest. 2004; 28: 101–104. 16760635

[10] Flower JE, Allender MC, Giovanelli RP, Summers S, Spoon TR, Leger JAS, et al Circulating concentrations of thyroid hormone in Beluga whales (*Delphinapterus Leucas*): influence of age, sex, and season. J Zoo Wildl Med. 2015; 46: 456–467. 10.1638/2014-0127.1 26352948

[11] Polat H, Dellal G, Baritci I, Pehlivan E. Changes of thyroid hormones in different physiological periods in white goats. J Anim Plant Sci. 2014; 24: 445–449.

[12] Olifiers N, Jansen AM, Herrera HM, de Cassia Bianchi R, D’Andrea PS, de Miranda Mourão G, et al Co-infection and wild animal health: effects of Trypanosomatids and gastrointestinal parasites on coatis of the Brazilian Pantanal. PLoS One. 2015; 10: e0143997 10.1371/journal.pone.0143997 26657699 PMC4678147

[13] Khalil B, Khodadad M, Pourjafar M, Mohsen G, Ebadolah M. Serum thyroid hormones and trace element concentrations in crossbred holstein cattle naturally infected with *Theileria annulata*. Comp Clin Pathol. 2011; 20: 115–120.

[14] Chethan GE, Garkhal J, De UK. Disturbance of thyroid function in canine ehrlichiosis and babesiosis associated with oxidative stress. Comp Clin Pathol. 2016; 25: 987–992.

[15] Kyriacou A, McLaughlin J, Syed A A. Thyroid disorders and gastrointestinal and liver dysfunction: A state of the art review. Eur J Intern Med. 2015; 26: 563–571. 10.1016/j.ejim.2015.07.017 26260744

[16] Yang Q, Meng X, Xia L, Feng Z. Conservation status and causes of decline of musk deer (*Moschus* spp.) in China. Biol Conserv. 2003; 109: 333–342.

[17] Sheng HL, Liu ZX. Musk deer population in China–rise and decline In: Ma YQ, editor. The Musk Deer in China. Shanghai: Shanghai Scientific & Technical Publishers; 2007 pp. 188–192.

[18] He L, Li LH, Wang WX, Liu G, Liu SQ, Liu WH, et al Welfare of farmed musk deer: changes in the biological characteristics of musk deer in farming environments. Appl Anim Behav Sci. 2014; 156: 1–5.

[19] Hu XL, Liu G, Wang WX, Zhou R, Liu SQ, Li LH, et al Methods of preservation and flotation for the detection of nematode eggs and coccidian oocysts in faeces of the forest musk deer. J Helminthol. 2016; 90: 680–684. 10.1017/S0022149X15000942 26560197

[20] Keech AL, Rosen DAS, Booth RK, Trites AW, Wasser SK. Fecal triiodothyronine and thyroxine concentrations change in response to thyroid stimulation in Steller sea lions (*Eumetopias jubatus*). Gen Comp Endocrinol. 2010; 166: 180–185. 10.1016/j.ygcen.2009.11.014 19941866

[21] Wasser SK, Azkarate JC, Booth RK, Hayward L, Hunt K, Ayres K, et al Non-invasive measurement of thyroid hormone in feces of a diverse array of avian and mammalian species. Gen Comp Endocrinol. 2010; 168: 1–7. 10.1016/j.ygcen.2010.04.004 20412809

[22] Hassanin A, Douzery EJ. Molecular and morphological phylogenies of Ruminantia and the alternative position of the Moschidae. Syst Biol. 2003; 52: 206–228. 12746147 10.1080/10635150390192726

[23] Cringoli G, Rinaldi L, Veneziano V, Capelli G, Scala A. The influence of floatation solution, sample dilution and the choice of McMaster slide area (volume) on the reliability of the McMaster technique in estimating the faecal egg counts of gastrointestinal strongyles and *Dicrocoelium dendriticum* in sheep. Vet Parasitol. 2004; 123: 121–131. 10.1016/j.vetpar.2004.05.021 15265576

[24] Bush AO, Lafferty KD, Lotz JM, Shostak AW. Parasitology meets ecology on its own terms: Margolis et al. revisited. J Parasitol. 1997; 83: 575–583. 9267395

[25] St Aubin DJ, Ridgway SH, Wells RS, Rhinehart H. Dolphin thyroid and adrenal hormones: circulating levels in wild and semidomesticated *Tursiops truncatus*, and influence of sex, age, and season. Mar Mamm Sci. 1996; 12: 1–13.

[26] Roth GS, Handy AM, Mattison JA, Tilmont EM, Ingram DK, Lane MA. Effects of dietary caloric restriction and aging on thyroid hormones of Rhesus monkeys. Horm Metab Res. 2002; 34: 378–382. 10.1055/s-2002-33469 12189585

[27] Petkov P, Kanakov D, Stoyanchev K. Quantitative variations in thyroid hormones-T3 and T4 in pigs of various breeds, gender and age. Trakia J Sci. 2008; 6: 16–20.

[28] Eshratkhah B, Sadaghian M, Eshratkhah S, Pourrabbi S, Najafian K. Relationship between the blood thyroid hormones and lipid profile in Moghani sheep: influence of age and sex. Comp Clin Pathol. 2010; 19: 15–20.

[29] Nicassio M, Aiudi G, Silvestre F, Matarrese R, Salvati ADS, Lacalandra GM. Free thyroid hormone and cortisol levels in stallions during the breeding season. Anim Reprod Sci. 2008; 107: 335–336.

[30] Bianco AC, Kim BW. Deiodinases: implications of the local control of thyroid hormone action. J Clin Invest. 2006; 116: 2571–2579. 10.1172/JCI29812 17016550 PMC1578599

[31] Fukuhara K, Kvetnansky R, Cizza G, Pacak K, Ohara H, Goldstein DS, et al Interrelations between sympathoadrenal system and hypothalamo–pituitary—adrenocortical / thyroid systems in rats exposed to cold stress. J Neuroendocrinol. 1996; 8: 533–541. 8843022 10.1046/j.1365-2826.1996.04877.x

[32] Da Costa VM, Moreira DG, Rosenthal D. Thyroid function and aging: gender-related differences. J Endocrinol. 2001; 171: 193–198. 11572803 10.1677/joe.0.1710193

[33] Suzuki S, Nishio SI, Takeda T, Komatsu M. Gender-specific regulation of response to thyroid hormone in aging. Thyroid Res. 2012; 5: 1–8. 10.1186/1756-6614-5-1 22280879 PMC3281778

[34] Hubl W, Schmieder J, Gladrow E, Demant T. Reference intervals for thyroid hormones on the Architect analyser. Clin Chem Lab Med. 2002; 40: 165–166. 10.1515/CCLM.2002.028 11939490

[35] West KL, Ramer J, Brown JL, Sweeney J, Hanahoe EM, Reidarson T, et al Thyroid hormone concentrations in relation to age, sex, pregnancy, and perinatal loss in bottlenose dolphins (*Tursiops truncatus*). Gen Comp Endocrinol. 2014; 197: 73–81. 10.1016/j.ygcen.2013.11.021 24321177

[36] Ingole SD, Deshmukh BT, Nagvekar AS, Bharucha SV. Serum profile of thyroid hormones from birth to puberty in buffalo calves and heifers. J Buffalo Sci. 2012; 1: 39–49.

[37] Osterman Lind E, Hoglund J, Ljungstrom BL, Nilson O, Uggla A. A field survey on the distribution of strongyle infections of horses in Sweden and factors affecting faecal egg counts. Equine Vet J. 1999; 31: 68–72. 9952332 10.1111/j.2042-3306.1999.tb03793.x

[38] Sol D, Jovani R, Torres J. Parasite mediated mortality and host immune response explains age-related differences in blood parasitism in birds. Oecologia. 2003; 135: 542–547. 10.1007/s00442-003-1223-6 16228253

[39] van Oers K, Richardson DS, Sather SA, Komdeur J. Reduced blood parasite prevalence with age in *Seychelles warbler*: selective mortality or suppression of infection? J Ornithol. 2010; 151: 69–77.

[40] Kuzmina TA, Dzeverin I, Kharchenko VA. Strongylids in domestic horses: Influence of horse age, breed and deworming programs on the strongyle parasite community. Vet Parasitol. 2016; 227: 56–63. 10.1016/j.vetpar.2016.07.024 27523938

[41] Klei TR, Chapman MR. Immunity in equine cyathostome infections. Vet Parasitol. 1999; 85: 123–136. 10485359 10.1016/s0304-4017(99)00093-x

[42] Herbert DR, Lee JJ, Lee NA, Nolan TJ, Schad GA, Abraham D. Role of IL-5 in innate and adaptive immunity to larval *Strongyloides stercoralis* in mice. J Immunol. 2000; 165: 4544–4551. 11035095 10.4049/jimmunol.165.8.4544

[43] Pit DSS, Polderman AM, Baeta S, Schulz-Key H, Soboslay PT. Parasite-specific antibody and cellular immune responses in humans infected with *Necator americanus* and *Oesophagostomum bifurcum*. Parasitol Res. 2001; 87: 722–729. 11570557 10.1007/s004360100419

[44] Matijatko V, Kis I, Torti M, Brkljacic M, Kucer N, Baricrafaj R, et al Septic shock in canine babesiosis. Vet Parasitol. 2009; 162: 263–270. 10.1016/j.vetpar.2009.03.011 19345507

