# Supplementary material for: Effects of breeding center, age and parasite burden on fecal triiodothyronine levels in forest musk deer
Source: PLoS One. 2018 Oct 1;13(10):e0205080. doi: 10.1371/journal.pone.0205080 (PMC6166975; doi:10.1371/journal.pone.0205080)
Supplement: S1 Table — Numbers in the brackets represent the number of valid data after removing the outliers. (DOCX) [file pone.0205080.s001.docx]

**S1 Table**

| Region | Female | | | |  | Male | | | |
| --- | --- | --- | --- | --- | --- | --- | --- | --- | --- |
|  | Age 3 | Age 5 | Age 7 | age9 |  | Age 3 | Age 5 | Age 7 | Age 9 |
| Shaanxi | 10(73) | 14(91) | 10(71) | 10(67) |  | 13(97) | 15(111) | 19(137) | 10(71) |
| Sichuan | 20(138) | 12(86) | 10(68) | 10(67) |  | 29(216) | 19(141) | 10(72) | 10(71) |
