# Supplementary material for: Effects of breeding center, age and parasite burden on fecal triiodothyronine levels in forest musk deer
Source: PLoS One. 2018 Oct 1;13(10):e0205080. doi: 10.1371/journal.pone.0205080 (PMC6166975; doi:10.1371/journal.pone.0205080)
Supplement: S2 Table — The significances were determined using the independent-samples t-test. (DOCX) [file pone.0205080.s002.docx]

**S2 Table**

| Breeding center | Age  (year) | Lactation  (Yes/No) | Individuals(data) | T3 (Mean ± SE; ng/g) | Significance |
| --- | --- | --- | --- | --- | --- |
| Shaanxi | 3 | Yes | 6 (43) | 133.77 ± 4.65 | t = 0.23  p = 0.83 |
|  |  | No | 4 (30) | 135.63 ± 7.26 |  |
|  | 5 | Yes | 7 (49) | 121.15 ± 4.33 | t = 0.62  p = 0.55 |
|  |  | No | 7 (42) | 124.50 ± 3.26 |  |
|  | 7 | Yes | 5 (36) | 111.58 ± 2.44 | t = 0.19  p = 0.86 |
|  |  | No | 5 (35) | 112.08 ± 0.96 |  |
|  | 9 | Yes | 5 (34) | 100.80 ± 0.36 | t = 1.37  p = 0.21 |
|  |  | No | 5 (33) | 101.80 ± 0.63 |  |
| Sichuan | 3 | Yes | 12 (85) | 146.65 ± 1.96 | t = 1.58  p = 0.13 |
|  |  | No | 8 (58) | 142.25 ± 1.72 |  |
|  | 5 | Yes | 8 (56) | 131.66 ± 2.99 | t = 2.10  p = 0.06 |
|  |  | No | 4 (28) | 139.35 ± 2.12 |  |
|  | 7 | Yes | 5 (38) | 132.26 ± 4.43 | t = 0.12  p = 0.91 |
|  |  | No | 5 (37) | 131.40 ± 6.04 |  |
|  | 9 | Yes | 5 (35) | 121.15 ± 7.47 | t = 0.58  p = 0.58 |
|  |  | No | 5 (32) | 127.51 ± 8.09 |  |
