# Supplementary material for: Effects of breeding center, age and parasite burden on fecal triiodothyronine levels in forest musk deer
Source: PLoS One. 2018 Oct 1;13(10):e0205080. doi: 10.1371/journal.pone.0205080 (PMC6166975; doi:10.1371/journal.pone.0205080)
Supplement: S3 Table — The significances were determined using the one-way ANOVA. (DOCX) [file pone.0205080.s003.docx]

**S3 Table**

| Breeding center | Age (year) | Female (F = 30.55) | | |  | Male (F = 28.59) | | |
| --- | --- | --- | --- | --- | --- | --- | --- | --- |
|  |  | 3 | 5 | 7 |  | 3 | 5 | 7 |
| Shaanxi | 5 | p = 0.122 |  |  |  | p = 0.001 |  |  |
|  | 7 | p = 0.001 | p = 0.008 |  |  | p < 0.001 | p < 0.001 |  |
|  | 9 | p < 0.001 | p < 0.001 | p < 0.001 |  | p < 0.001 | p < 0.001 | p = 0.025 |
| Sichuan | 5 | p = 0.005 |  |  |  | p = 0.002 |  |  |
|  | 7 | p < 0.001 | p = 0.003 |  |  | p < 0.001 | p = 0.010 |  |
|  | 9 | p < 0.001 | p < 0.001 | p < 0.001 |  | p < 0.001 | p < 0.001 | p = 0.044 |
